# Brain cannabinoid CB1 receptor signaling modulates reward responses and inhibitory control in humans

**DOI:** 10.1162/IMAG.a.1344

**Published:** 2026-08-26

**Authors:** Lihua Sun, Tatu Kantonen, Laura Pekkarinen, Pirjo Nuutila, Lauri Nummenmaa

**Affiliations:** Huashan Institute of Medicine, Huashan Hospital, Fudan University, Shanghai, China; Department of Psychiatry, Huashan Hospital, Fudan University, Shanghai, China; Turku PET Centre, University of Turku, Turku, Finland; Turku PET Centre, Turku University Hospital, Turku, Finland; Turku Collegium for Science, Medicine and Technology, University of Turku, Turku, Finland; Department of Neurosurgery, Helsinki University Hospital, Helsinki, Finland; Endocrinology, Abdominal Center, Helsinki University Hospital, Helsinki, Finland; Department of Endocrinology, Turku University Hospital, Turku, Finland; Department of Psychology, University of Turku, Turku, Finland

**Keywords:** appetite, reward, inhibition, PET, fMRI

## Abstract

The central endocannabinoid system, particularly the *in vivo* cannabinoid type 1 (CB1) receptor signaling, presents a promising target for treating eating disorders. However, its precise role in appetite control remains unclear. This study aimed to determine how CB1 receptor signaling contributes to key aspects of appetite regulation, specifically anticipatory food reward responses and inhibitory control. Forty-one healthy male participants underwent [^18^F]FMPEP-d2 positron emission tomography (PET) to quantify CB1 receptor availability. Functional magnetic resonance imaging (fMRI) was used to assess anticipatory neural responses to food cues, while inhibitory control was measured using a go/nogo task. Data show that individuals with higher CB1 receptor availability exhibited stronger anticipatory reward-related neural responses and reduced activation during inhibitory control. Therefore, CB1 receptor signaling plays a distinct role in modulating reward and inhibitory processes related to feeding behavior. The findings suggest that the CB1 receptor may serve as a therapeutic target for regulating appetite.

## 1 Introduction

Overeating and obesity are significant global health concerns. Obesity is associated with an imbalance in brain systems governing appetite control, in that the reward circuit is overactive to reward anticipation and inhibitory networks may fail to engage control of the reward circuit (Volkow et al., 2008). The interaction between food-evoked reward responses and inhibitory control is crucial in understanding appetite regulation, since aberrant function of these systems may lead to overeating and weight gain (Appelhans et al., 2011). The molecular mechanisms underlying appetite control are not fully understood, but various neurotransmitter pathways, such as the dopamine and opioid signaling, are central to feeding (Berridge, 2009; Nummenmaa et al., 2018). Accumulating evidence also points to a key role for the endocannabinoid system in regulating appetite (D’Addario et al., 2014). This is underscored by the initial success of the CB1 receptor antagonist rimonabant in managing weight (Van Gaal et al., 2005), although its clinical use was discontinued due to psychiatric side effects (Di Marzo & Després, 2009). Marijuana use may stimulate appetite (Foltin et al., 1986), but its long-term effects seem to lower the likelihood of overeating in users (Hayatbakhsh et al., 2010; Le Strat & Le Foll, 2011). The role of endocannabinoid signaling in feeding behavior is also highlighted by preclinical evidence showing that cerebral CB1 receptor knockout leads to inhibited hedonic feeding (Ruiz de Azua et al., 2021), while edible cannabinoids stimulating CB1 receptor signaling may reduce inhibitory control and promote impulsive eating behavior (Lord et al., 2025). In our recent study involving generally healthy individuals, we further showed that family risk factors for obesity, exercise habits, and body mass indices are all potential modulators of central and peripheral CB1 receptor signaling (Kantonen et al., 2022).

However, the impact of endocannabinoid signaling on brain function and eating behavior is complex, and its exact role in brain cognitive functions, especially in those relevant for appetite control, remains unresolved. This complexity presents both therapeutic potential and challenges, especially for developing clinical interventions targeting eating disorders (Cristino et al., 2020; D’Addario et al., 2014). Elevated levels of the endocannabinoid 2-arachidonoyl glycerol (2-AG) in obesity correlate with higher body fat and fasting insulin levels, indicating a link between the endocannabinoid system and metabolic dysregulation (Bluher et al., 2006). Preclinical studies show that CB1 receptor activation promotes eating, supporting that CB1 receptor antagonists could help curb overeating (Aguilera Vasquez & Nielsen, 2022; Van Gaal et al., 2005). Nevertheless, understanding CB1 receptor signaling in appetite control is difficult due to its interactions with other neurotransmitter systems, such as the dopamine and opioid pathways (Polissidis et al., 2013). For example, activation of CB1 receptors affects the mesolimbic dopamine system, which governs reward and reinforcement behaviors, positioning CB1 receptor as potentially substitutive for food intake by triggering dopamine-related satiation effects. This intricate receptor interaction contributes to the current ambiguity surrounding CB1 receptor’s role in food reward responses. Despite some evidence suggesting cannabis use may protect against obesity, findings remain mixed, with discussions on acute versus long-term effects (Hayatbakhsh et al., 2010; Le Strat & Le Foll, 2011). Advances in differentiating the roles of CB1, dopamine, and opioid receptors could yield new insights into managing eating behaviors and disorders through targeted pharmacological pathways.

In the current study, we investigated the specific role of central CB1 receptor signaling and its impact on appetitive processes, specifically the reward response and inhibitory control where aberrant functions may reflect the two common culprits for overeating. CB1 receptor availability was quantified using positron emission tomography (PET) and radioligand [^18^F]FMPEP-d2 which binds selectively to CB1 receptors (Donohue et al., 2008; Terry et al., 2010). Acute responses to anticipatory food reward as well as inhibitory control were measured using task-based fMRI. We hypothesize that higher CB1 receptor availability is associated with enhanced appetite, as is to be illustrated by increased anticipatory food-reward responses and reduced inhibitory control.

## 2 Materials and Methods

### 2.1 Participants

Healthy male participants with age of 20–35 years and BMI of 18.5–30 kg/m^2^ were studied with fMRI (n = 41) and PET (37 out of 41) measures. None of the participants had detectable levels of 11-Nor-9-carboxy-delta9-tetrahydrocannabinol in their blood (a marker of cannabis consumption). Exclusion criteria include smoking or use of nicotine products, abusive use of alcohol, use of illicit drugs, any chronic disease or medication that could affect glucose metabolism or neurotransmission, neurological or psychiatric disease, eating disorder, any contraindication to magnetic resonance imaging (MRI) and prior participation in PET studies or other significant exposure to radiation. The study protocol was approved by the Ethics Committee of the Hospital District of Southwest Finland and conducted in accordance with the principles of the Declaration of Helsinki. Written informed consent was obtained from all participants prior to inclusion. The clinical investigation was registered at clinicaltrials.gov (Neuromolecular Risk Factors for Obesity, PROSPECT, NCT03106688), and sample characteristics have been presented previously (Kantonen et al., 2022). The brain-PET data relating to CB1 receptors and obesity risk have been reported previously (Kantonen et al., 2022), whereas the current study examines the association between CB1 receptor signaling and previously unreported fMRI data on anticipatory reward and inhibitory control.

### 2.2 PET data acquisition

Participants fasted for at least 6 hours before the [^18^F]FMPEP-d2 PET scans and were instructed to *refrain from* consuming caffeinated beverages, alcohol or tobacco, and from engaging physical exercise on the day of the PET scan as well as the day before. Estimates of cerebral CB1 receptor density with radioligand [^18^F]FMPEP-d2 have demonstrated excellent test-retest reliability with intraclass correlation coefficient around 0.83 (Barros et al., 2014). PET images were obtained using a PET/CT scanner (GE Discovery VCT, GE Healthcare), and all scans took place between 12:00 and 16:00 hours. The tracer was administered via a catheter inserted into the antecubital vein, with a targeted dose of 185 MBq. The brain’s radioactivity was followed for 60 minutes using the following framing schedule: 3 × 60 seconds, 5 × 180 seconds, and 7 × 360 seconds. To prevent excessive head movement, the participants’ heads were secured to the scan table. CT scans were performed prior to the PET scans for attenuation correction. Throughout the scanning process, participants were asked to rest and were monitored by a physician. Plasma radioactivity was assessed at regular intervals using arterialized blood samples, measured with an automatic gamma counter (Wizard 1480 3”, Wallac, Turku, Finland). T1-weighted anatomical MR images (TR: 8.1 ms; TE: 3.7 ms; flip angle: 7°; scan duration: 263 seconds; voxel size: 1 mm³ isotropic) were acquired with a PET/MR scanner (Ingenuity TF PET/MR, Philips) for anatomical normalization and reference.

### 2.3 PET data modelling

The PET data were processed using the automated tool Magia (Karjalainen et al., 2020). The processing pipeline begun with motion correction of the PET scans, followed by co-registration of the PET and MR images. Before computing the parametric images, the data was smoothed with a Gaussian kernel (FWHM = 6 mm) to improve the signal-to-noise ratio for model fitting. Finally, parametric images were spatially normalized to MNI space and smoothed again using a Gaussian kernel (FWHM = 6 mm). CB1 receptor availability was quantified as [18F]FMPEP-d2 volume of distribution (VT) using graphical analysis (Logan) (Logan, 2000), where the starting point of 36 minutes was used and plasma activities were corrected for metabolites (Kantonen et al., 2022; Lahesmaa et al., 2018).

### 2.4 fMRI data acquisition

MRI data were collected using the Phillips Ingenuity TF PET/MR 3T whole-body scanner. High-resolution structural brain images (1 mm³) were obtained using a T1-weighted sequence. Functional MRI (fMRI) data were acquired with a T2*-weighted echo-planar imaging sequence (TR = 2600 ms, TE = 30 ms, 75° flip angle, 240 mm field of view, 80 × 80 reconstruction matrix, 62.5 kHz bandwidth, 3.0 mm slice thickness, 45 interleaved slices acquired in ascending order without gaps). For the inhibitory control task, 145 functional volumes were collected, and 165 volumes for the food-reward task. Both PET and fMRI measurements were acquired under overnight fasting conditions. Due to logistical considerations and radiotracer production schedules, PET and fMRI sessions for a given participant were not necessarily conducted on the same day, but were scheduled within a 1–3 week interval.

### 2.5 Behavioral measures

#### 2.5.1 Anticipatory food reward experiment

**Fig. 1.**
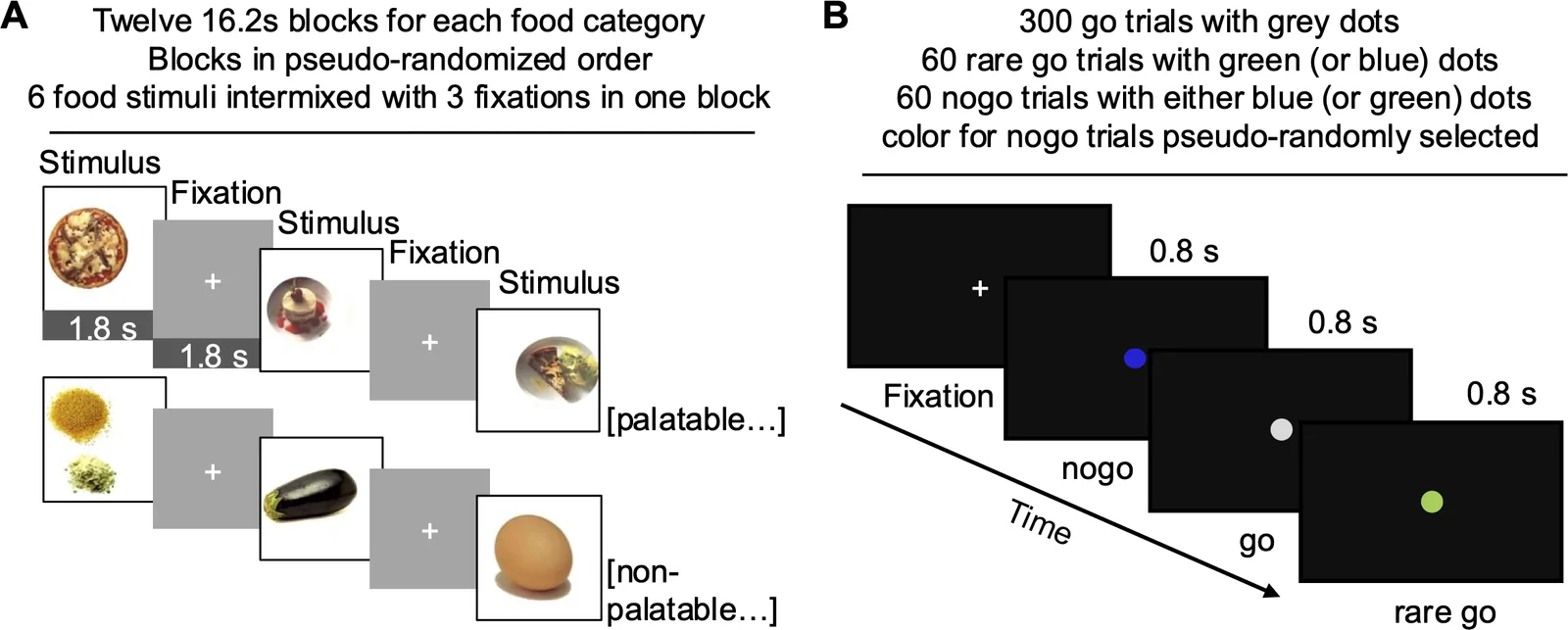
Behavioral tasks. (A) Design for the anticipatory food-reward experiment. (B) Design for the response inhibition task.

We employed a previously established task (Laurila et al., 2021; Nummenmaa et al., 2018) to induce anticipatory reward response by presenting participants with images of palatable foods (e.g., chocolate, pizza, cakes) and bland foods (e.g., lentils, cereal, eggs), Figure 1A. This task mimics real-life situations where appetite is stimulated by visual food cues, such as those found in advertisements. During the task, participants viewed alternating 16.2-second blocks displaying either palatable or bland food images. Each block contained nine images from one category, interspersed with fixation crosses. The food images were shown on either the left or right side of the screen, and participants were instructed to indicate the location by pressing corresponding buttons, ensuring they paid attention to the stimuli. Participants first completed the food-reward task, which was followed by the inhibitory control task. Behavioral tasks were administered using Presentation software (Neurobehavioral System, Inc., Berkeley, CA, USA).

#### 2.5.2 Go/nogo task for measuring inhibitory control task

Participants were instructed to press a button with their left hand in response to a “go” signal and to refrain from pressing the button during a “nogo” signal, Figure 1B. Small dots were displayed sequentially in the center of the computer screen at 0.8-second intervals. The dots appeared in three colors: gray (70% of trials), green (15%), and blue (15%). Gray dots always signaled “go,” requiring participants to press the button. Green and blue dots were randomly assigned as either rare “go” or “nogo” signals for each participant. Because the ‘go’ trials occur more frequently than the ‘nogo’ trials, withholding a response requires exerting inhibitory control. Successful task performance engages neural processes underlying sustained attention, motor execution, and response inhibition. The statistical contrast for inhibitory activation was based on equal probability of the green and blue dots serving as signals.

### 2.6 fMRI data processing

MRI data were processed using fMRIPrep version 1.3.0.2 (Esteban et al., 2019). Structural T1 images underwent correction for intensity non-uniformity, skull stripping, brain surface reconstruction, and spatial normalization to the ICBM 152 Nonlinear Asymmetrical template version 2009c through nonlinear registration using antsRegistration (ANTs 2.2.0) and brain tissue segmentation. Functional MRI data were processed through a series of steps: co-registration with the T1 reference image, slice-time correction, spatial smoothing with a 6 mm Gaussian kernel, and automatic removal of motion artifacts using ICA-AROMA. The data were then resampled to the MNI152NLin2009cAsym standard space. The quality of the images was visually inspected to ensure whole-brain field of view coverage, proper alignment with the anatomical images, and absence of signal artifacts, and this was further verified through visual reports generated by fMRIPrep. All functional data were included in the present study.

### 2.7 Statistical analysis

#### 2.7.1 Behavioral data

Reaction times for the go trials were analyzed, with outlier values below 100 ms or above 800 ms removed, as previously (Sun et al., 2023). Reaction times were analyzed separately using a mixed-effects linear model, with mean whole-brain GM CB1 receptor Availability, Age and BMI as fixed factors and Subject as a random factor. Accuracy rates were calculated as the percentage of “no response” in all nogo trials, and they were analyzed using linear regression model with GM CB1 Receptor Availability, Age and BMI as factors. All analyses were conducted using R statistical software. Button responses in the food reward task were only acquired to ensure attention to the stimuli and were consequently not analyzed.

#### 2.7.2 fMRI data

The full-volume fMRI data were analyzed in SPM12. The whole-brain random effects model was applied using a two-stage process with separate first and second levels. The food-reward task used a mixed block and event-related design, with blocks of same-category food images presented at random intervals and analyzed using an event-related model. The go/nogo task was run an event-related design and analyzed accordingly. For each subject, first-level GLM was used to predict regional effects of task parameters on BOLD indices of activation. These parameters are in the response inhibition task: nogo vs. go signals; go signals include both “go” and “rare go” signals; in the food-reward experiment, palatable vs. bland food pictures. First-level contrast images were then subjected to second-level analysis. Statistical maps were thresholded at a voxel-wise threshold of p < 0.05 (uncorrected), and cluster-level significance was determined using FDR correction at p < 0.05.

#### 2.7.3 PET-fMRI fusion analysis

To map the association between cerebral CB1 receptor and anticipatory reward and inhibition responses, we used PET-fMRI fusion analyses where hemodynamic responses to the i) palatable vs. bland food images and ii) inhibitory responses (nogo vs. go trials). Because high regional autocorrelation of CB1 receptor availability (r > 0.9, Supplementary Fig. S1) mean grey matter CB1 availability (rather than regional availabilities) was used to predict task-specific response in the fMRI data, while controlling for Age and BMI. Specifically, mean grey matter CB1 receptor availability, age, and BMI were entered as regressors to predict the haemodynaic responses (first-level contrast images) corresponding to reward-related and inhibitory-control-related responses. Statistical threshold was set at p < 0.05 with FDR correction at cluster level.

#### 2.7.4 Regions of interest analysis

Along with full-volume analysis, brain regional BOLD and CB1 receptor availabilities were analyzed with Pearson correlation analysis. Eight regions of interest (ROIs) were selected including the anterior cingulate cortex (ACC), amygdala, caudate, middle frontal cortex (MFC), insula, posterior cingulate cortex (PCC), putamen, and thalamus. The ROIs were selected based on their established roles in cognitive functions related to reward processing and inhibitory control (Laurila et al., 2021; Sun et al., 2023). ROI analyses are conducted primarily for visualization, given the limited existing knowledge regarding the specific regional functions of CB1 receptor signaling.

## 3 Results

### 3.1 Brain CB1 receptor availability

**Fig. 2.**
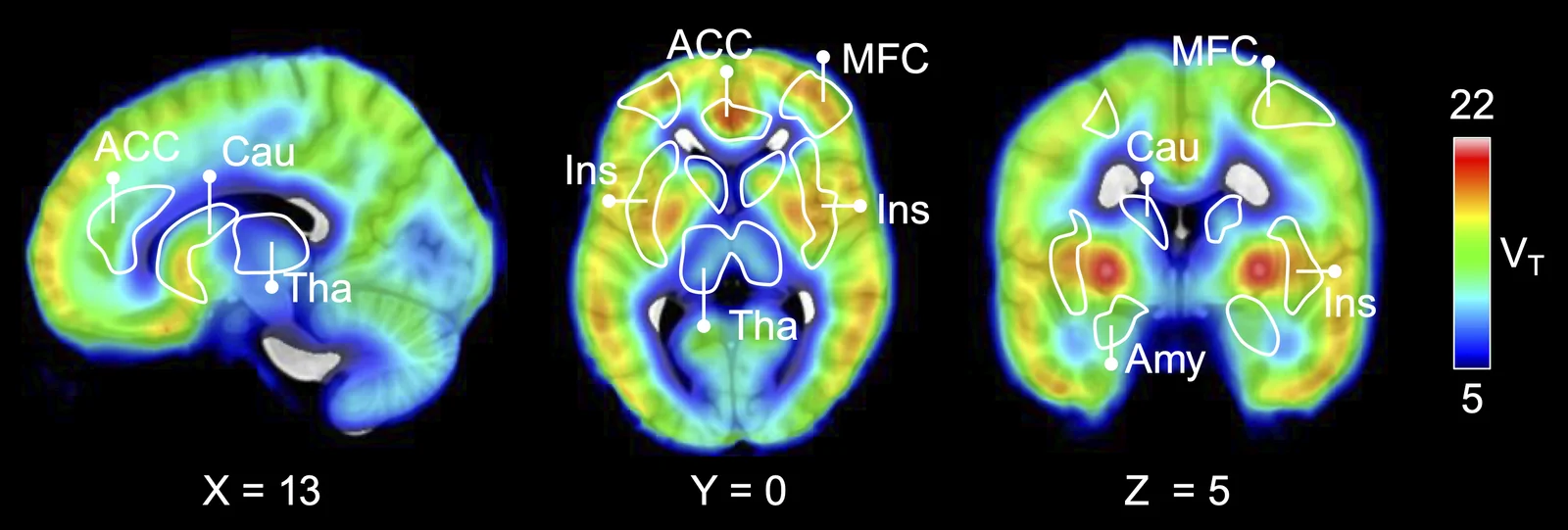
Mean volume of distribution (V_T_) of brain CB1 receptor availability in the sample. Regions of interest are labeled. ACC = anterior cingulate cortex, Amy = amygdala, Cau = caudate, Ins = insula, MFC = middle frontal cortex, PCC = posterior cingulate cortex, Put = putamen, Tha = thalamus. Correlations between regional values of receptor availability are shown in Supplementary Figure S1.

Mean distribution of brain CB1 receptors is shown in Figure 2. CB1 receptor availabilities in the selected ROIs are found in Supplementary Table S1.

### 3.2 CB1 receptor availability and brain food-reward responses

**Fig. 3.**
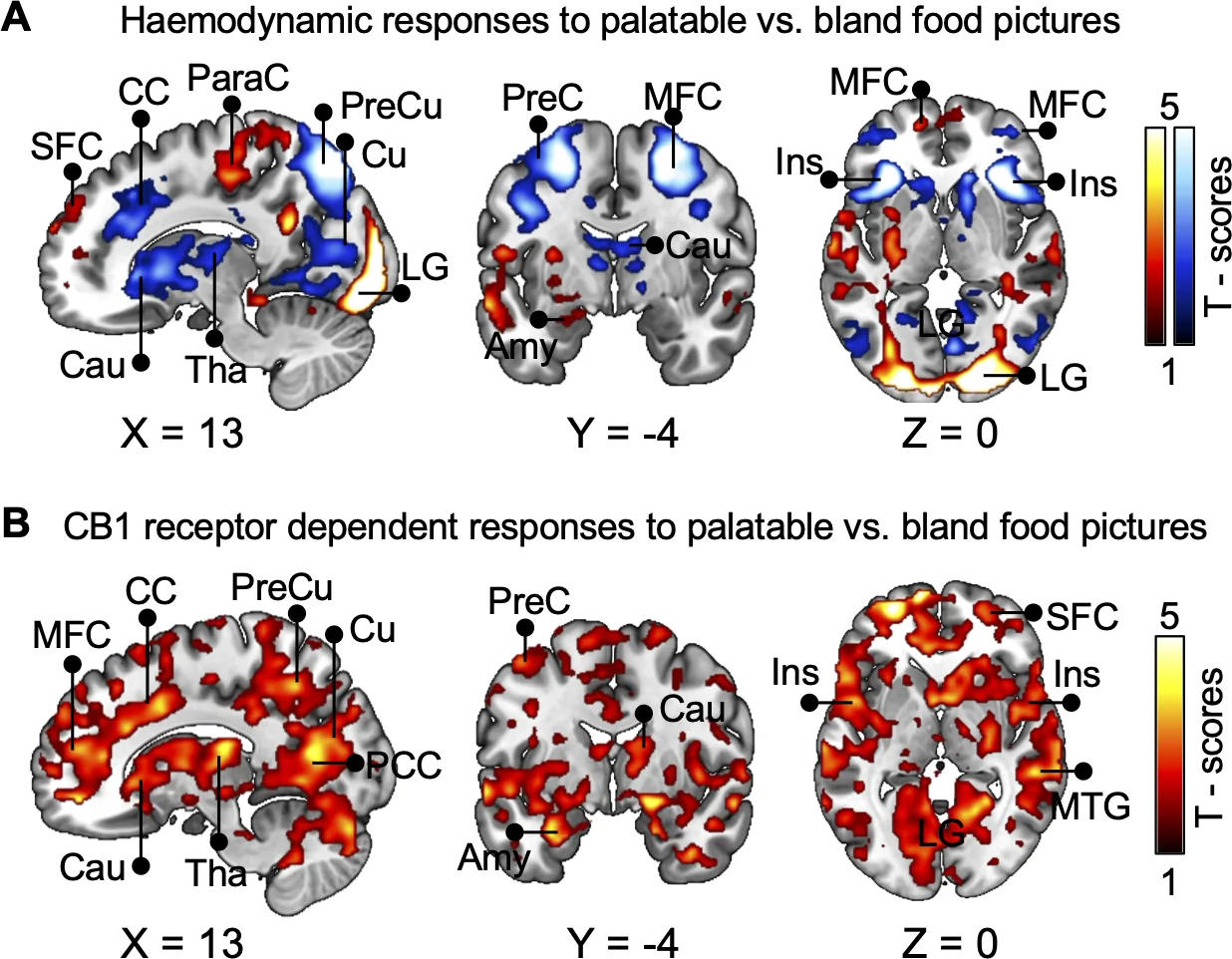
Hemodynamic responses to palatable food images and their dependency on CB1 availability. (A) Hemodynamic BOLD responses to palatable versus bland food pictures. Hot colors indicate increased BOLD signals, while cold colors indicate reduced BOLD signals. (B) Increased CB1 receptor availability is associated with enhanced brain food-reward responses. Data are FDR-corrected at p < 0.05. Amy = amygdala, Cau = caudate, CC = cingulate cortex, Cu = cuneus, Ins = insula, LG = lingual gyrus, MFC = medial frontal cortex, MTG = middle temporal gyrus, ParaC = paracentral cortex, PCC = posterior cingulate cortex, PreC = precentral cortex, PreCu = precuneus, SFC = superior frontal cortex, Tha = thalamus.

In the anticipatory food-reward task, hemodynamic responses were elevated to palatable versus non-palatable food pictures in regions such as the paracentral area and lingual gyrus (Fig. 3A). In contrast, decreased neural responses to palatable versus non-palatable foods were observed in larger brain clusters including the caudate, thalamus, insula, precentral cortex, precuneus, cingulate cortex, and middle frontal cortex.

**Fig. 4.**
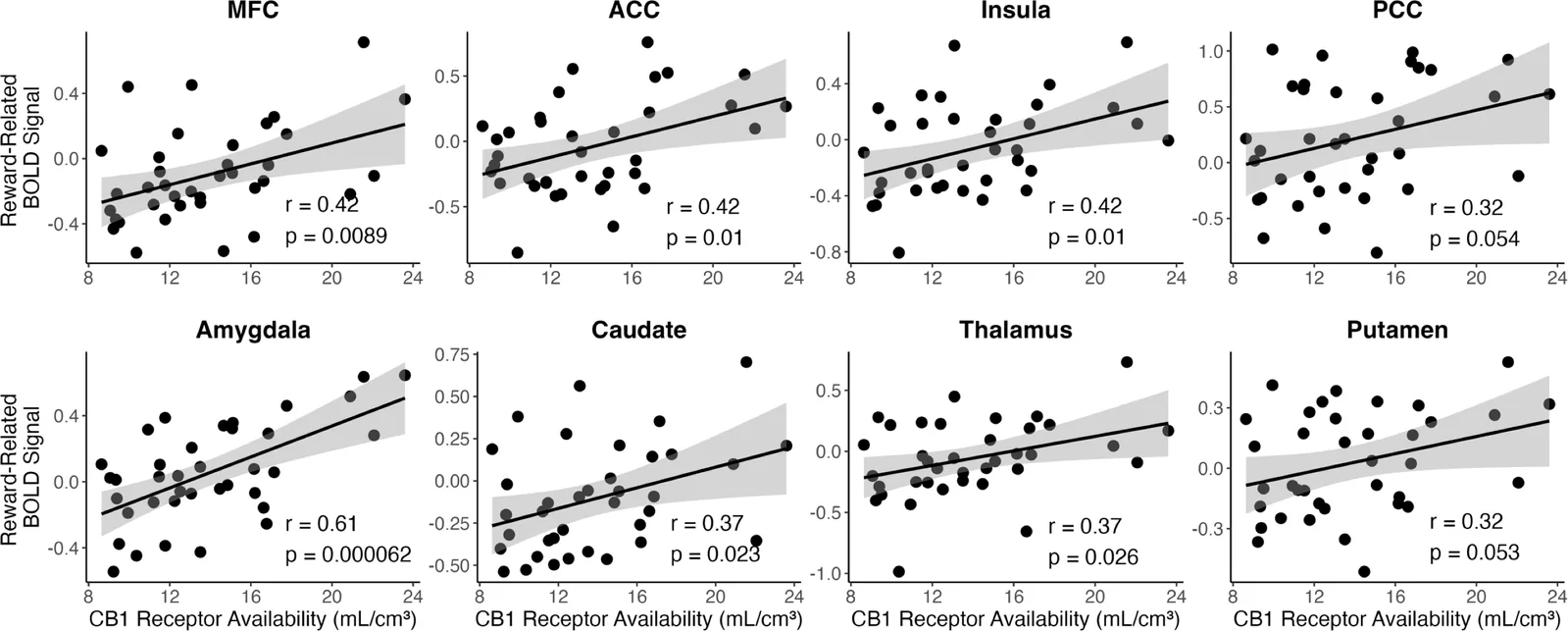
ROI-level correlations between grey matter CB1 availability and BOLD responses to palatable versus bland food images. ACC = anterior cingulate cortex; MFC = middle frontal cortex; PCC = posterior cingulate cortex.

In the PET-fMRI fusion analysis higher CB1 receptor availability was positively associated with increased hemodynamic responses to palatable versus non-palatable foods in large brain clusters spanning the caudate, thalamus, amygdala, insula, middle frontal cortex, cingulate cortex, precuneus, posterior cingulate cortex, mid-temporal gyrus, and lingual gyrus (Fig. 3B). The shape of these associations in selected anatomical regions is visualized in Figure 4. No negative associations were found between CB1 availability and the hemodynamic reward responses.

### 3.3 CB1 receptor availability and inhibitory control

**Fig. 5.**
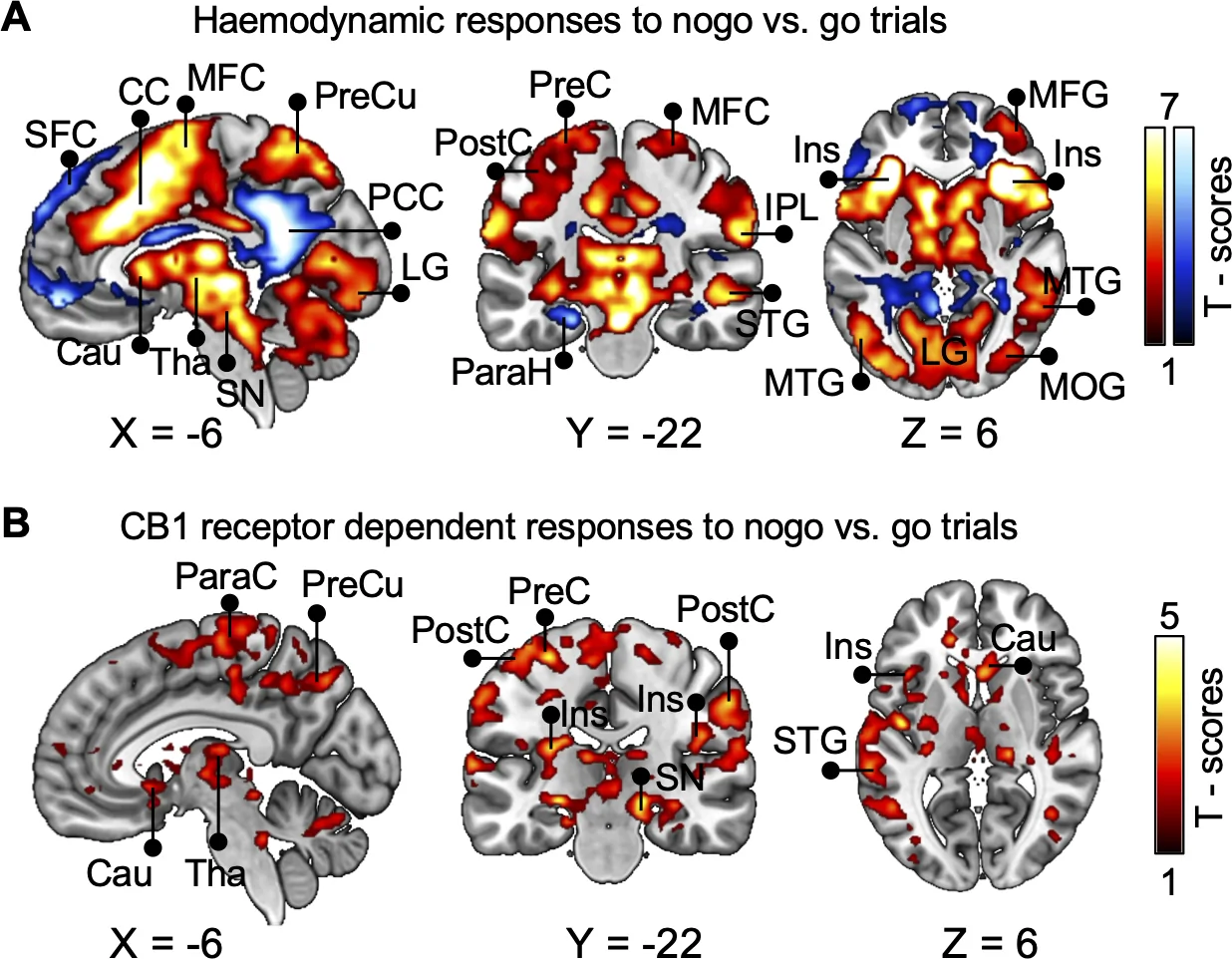
Hemodynamic responses to inhibition and their dependency on CB1 availability. (A) Increased (hot color) and decreased (cold color) hemodynamic responses during nogo versus go trials. (B) Increased CB1 receptor availability is associated with greater effort in inhibitory control. Data are FDR-corrected at p < 0.05. Cau = caudate, CC = cingulate cortex, Ins = insula, IPL = inferior parietal lobe, LG = lingual gyrus, MFC = middle frontal cortex, MOG = middle occipital gyrus, MTG = middle temporal gyrus, ParaC = paracentral cortex, ParaH = parahippocampus, PCC = posterior cingulate cortex, PreC = precentral cortex, PreCu = precuneus, SFC = superior frontal cortex, SN = substantia nigra, STG = superior temporal gyrus, Tha = thalamus.

Hemodynamic responses were larger during nogo versus go trials in large clusters encompassing the primary and secondary motor cortical areas (including the precentral cortex, postcentral cortex, and paracentral area), insula, caudate, thalamus, substantia nigra, middle frontal cortex, precuneus, inferior parietal lobe, superior temporal gyrus, mid-temporal gyrus, and mid-occipital gyrus (Fig. 5A). Reduced responses were also observed in regions such as the superior frontal gyrus and the posterior cingulate cortex.

**Fig. 6.**
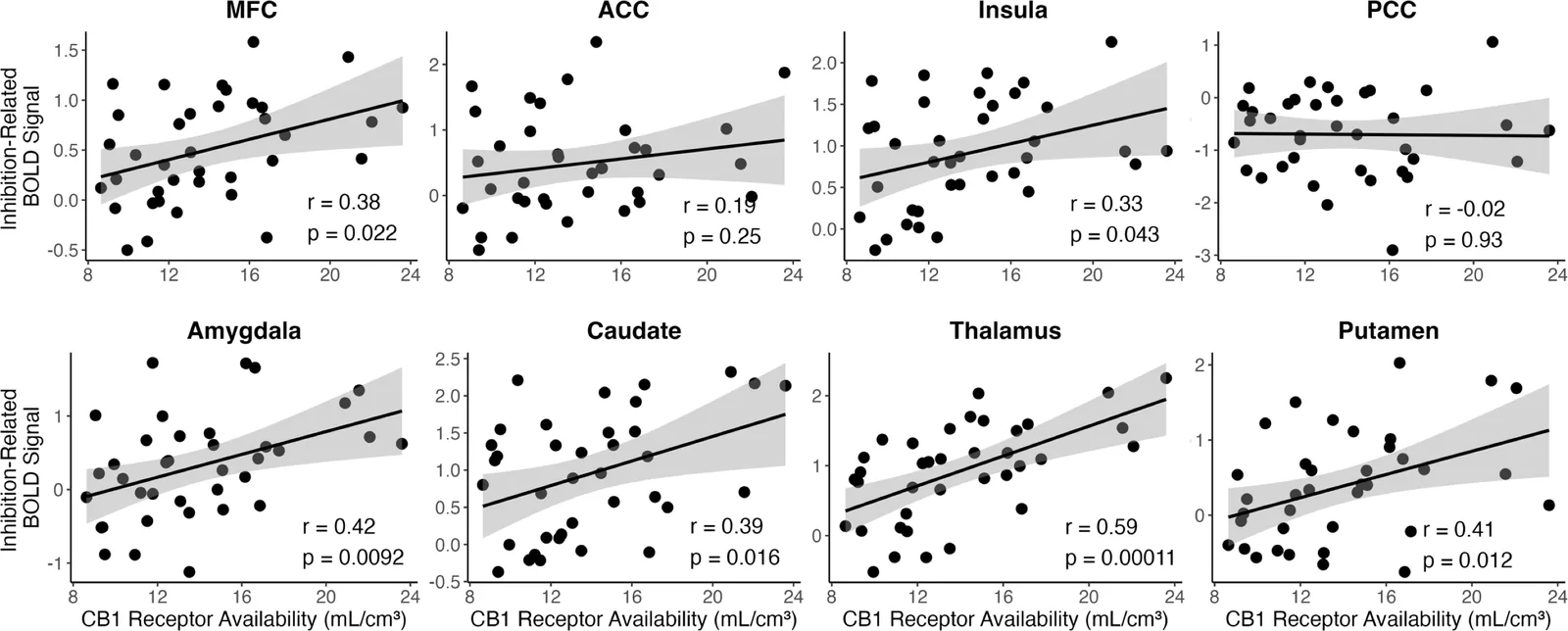
ROI-level correlations between grey matter CB1 availability and BOLD responses to nogo versus go trials. ACC = anterior cingulate cortex; MFC = middle frontal cortex; PCC = posterior cingulate cortex.

PET-fMRI fusion analysis indicated that increased CB1 receptor availability was associated with heightened activation in the primary and secondary motor areas, cuneus, thalamus, insula, precuneus, superior temporal gyrus, and substantia nigra (Fig. 5B). No negative associations were found between CB1 availability and BOLD responses during the inhibition task. The shape of these associations in selected anatomical regions is visualized in Figure 6.

These associations were however not manifested in the behavioral response data. CB1 availability had no statistically significant effects on reaction times either when “rare go” trails were included (β = -0.042, 95% CI [-0.097, 0.012]) or when they were excluded (β = -0.043, 95% CI [-0.097, 0.012]). Brain CB1 receptor availability also had no impact on accuracy rate in the response inhibition performance (β = -0.011, 95% CI [-0.032, 0.010]). Neither age nor BMI had statistically significant impact on either reaction times or accuracy rate.

## 4 Discussion

Our key finding was that the CB1 receptor-mediated endocannabinoid system is closely linked to brain reward responses and inhibitory control. Unlike traditional pharmaceutical studies where a specific drug is used to stimulate the endocannabinoid system to disclose its potential functions, the current study leverages the *in vivo* nature of PET imaging, leaving the targeted system unaffected and the focus is on mapping the CB1 receptors in their basal state. Cannabinoid receptor signaling is highly intertwined with the dopamine and opioid systems, making traditional studies insufficient in isolating the specific role of the cannabinoid CB1 receptor in brain functions. This study, therefore, bears merits in providing a clearer understanding of the specific role of this system. These findings supports previous studies showing that deactivating this pathway can reduce food intake (Van Gaal et al., 2005), while activation promotes weight gain (Andries et al., 2014). These data may thus indicate a modulatory effect of CB1 receptor signaling on appetite, and therefore supports therapeutic strategies targeting the endogenous cannabinoid system for treating eating disorders.

Normal-weight individuals exhibit widely diverse responses to palatable versus bland food images. Contrary to our previous findings showing dominant BOLD signal activation in response to palatable food images (Nummenmaa et al., 2018; Sun et al., 2023), participants in the current study displayed large-cluster suppression in reward circuits, including the caudate. This may suggest, at the sample level, a preference for bland light-tasting over high-caloric (assumed to be palatable) food images. However, we found that the up- or down-regulated preference of high-caloric food is genuinely explained by the individual levels of cannabinoid CB1 receptor availability. Higher CB1 receptor signaling is consistently associated with stronger responses to high-caloric food pictures, affecting neural activity in major reward circuits. This result thus suggests that the individual variation in the function in the appetitive circuits is dependent on the CB1 receptor availability; comparable findings have been found for the link between μ-opioid receptor system and food rewards (Nummenmaa et al., 2018).

Our data also show that individuals with higher CB1 receptor availability may exhibit compromised inhibitory control. This is demonstrated with increased effort in performing the task of inhibition, as reflected in increased BOLD signals in key brain nodes. The inhibitory control task is highly engaging, requiring high attentional concentration, accurate and rapid responses. Go/nogo task performance proves to be sensitive to endocrinological circumstances, where infusion of a satiation-inducing hormone reduces brain reward responses and enhances inhibitory control (Sun et al., 2023). Therefore, together with increased food-reward responses, the stronger engagement of cognitive control processes may contribute an enhanced appetite in participants with higher brain cannabinoid CB1 receptor availability, although this link needs to be validated in follow-up work. However, we note that these effects did not translate to behavioral performance in the task, suggesting that their magnitude is not sufficiently strong for influencing de facto outcomes in inhibitory processes measured in this type of simplified tasks.

Clinically, these findings have significant implications for understanding and managing eating disorders and obesity. Therapeutic strategies that modulate CB1 receptor activity could offer a targeted approach to restore balance between reward sensitivity and inhibitory control, as to be monitored via behavioral approaches. For instance, pharmacological interventions that block CB1 receptor signaling should efficiently attenuate the hyper-responsiveness to external food cues. Similarly, personalized treatments could consider individual variations in CB1 receptor availability to optimize behavioral or pharmacological interventions for weight management. These findings also emphasize the importance of integrating neuroimaging biomarkers into clinical research, enabling precise identification of individuals who might benefit from cannabinoid-based treatments. Overall, targeting the endogenous cannabinoid system offers a promising avenue for addressing the growing public health challenge posed by eating disorders and obesity.

Previous studies on CB1 receptor signaling have linked its functions with dopamine and opioid signaling pathways. For example, activating CB1 receptor leads to increased release of dopamine in the brain limbic regions (Polissidis et al., 2013), and also activating dopamine D2 receptors promote release of anandamide, a endocannabinoid (Giuffrida et al., 1999). Pharmaceutical effects are largely enhanced when targeting both receptor signaling pathways simultaneously (Glass & Felder, 1997), with findings showing that these two receptors form heterodimers in co-expressed subcortical regions (Kearn et al., 2005). The CB1 receptors also form heterodimer complex with the MORs in specific brain regions (Hojo et al., 2008), with functional overlap including modulating pain pathway and reward (Christie, 2006). This linkage between MORs and CB1 receptors challenges traditional pharmaceutical studies applying drug modulation of the neurotransmission system, making the specific role of CB1 receptor signaling ambiguous. In contrast, the current study highlighting a specific role of the cannabinoid systems bears neuroscientific merits.

### 4.1 Limitations

Our study included only non-obese males, limiting the generalizability of the findings to females or those with metabolic dysregulation. All measurements were conducted under an overnight fasting to minimize physiological variability. Further studies are thus needed to characterize the role of CB1 receptors in, for example, obesity and satiety. Additionally, we focused on acute brain responses to appetite-inducing images and inhibitory control, thus we cannot resolve how CB1 receptors would regulate responses to actual feeding or long-term-feeding behavior in general. Technically, while [^18^F]FMPEP-d2 binding in baseline condition is proportional to cannabinoid C1 receptor density, the exact contributions of receptor density, receptor affinity, and baseline occupancy by endogenous cannabinoid cannot be assessed in a single measurement, and these components cannot be differentiated in a single scan. Moreover, due to the high autocorrelation among regional measures of CB1 receptor availability, it is not feasible in the current study to efficiently disentangle the specific regional contributions of CB1 receptor signaling to cognitive functions. Finally, although we observed an association between baseline CB1 receptor availability and haemodynamic responses to reward and inhibition, both causality and the potential mediating mechanisms (e.g., network connectivity, downstream molecular signaling pathways) remain unresolved.

## 5 Conclusions

Brain cannabinoid CB1 receptor availability modulates reward responses and inhibitory control, key neural mechanisms involved in appetite regulation. The findings suggest that individuals with elevated CB1 receptor availability may require increased engagement of inhibitory control mechanisms to counteract reward-driven responses triggered by external cues, as opposed to enhanced internal satiety signals. These insights have potential implications for developing therapeutic strategies targeting the endogenous cannabinoid system to address obesity epidermic.

## Supplementary Material

Supplementary Material

## Data Availability

The current study is based on human subject PET–fMRI data. As per Finnish legislation, the medical imaging data are considered sensitive personal information and cannot be publicly shared even in anonymized format. Enquiries regarding the dataset can be sent to Lauri Nummenmaa by: email to latanu@utu.fi or post to Turku PET Centre c/o Turku University Hospital, Kiinamyllynkatu 4-8, FI-20520 Turku, Finland.
