# Supplementary material for: Brain cannabinoid CB1 receptor signaling modulates reward responses and inhibitory control in humans
Source: Imaging Neurosci (Camb). 2026 Aug 26;4:IMAG.a.1344. doi: 10.1162/IMAG.a.1344 (PMC13519986; doi:10.1162/IMAG.a.1344)
Supplement: Supplementary file 1 [file IMAG.a.1344_supp.pdf]

Supplementary Data

Brain Cannabinoid CB1 Receptor Signaling Modulates Reward Responses and Inhibitory Control in Humans

By Lihua Sun, et al.
Email: lihua.sun@utu.fi
Address: Turku PET Centre, Kiinamyllynkatu 4-8, Turku, 20520, Finland

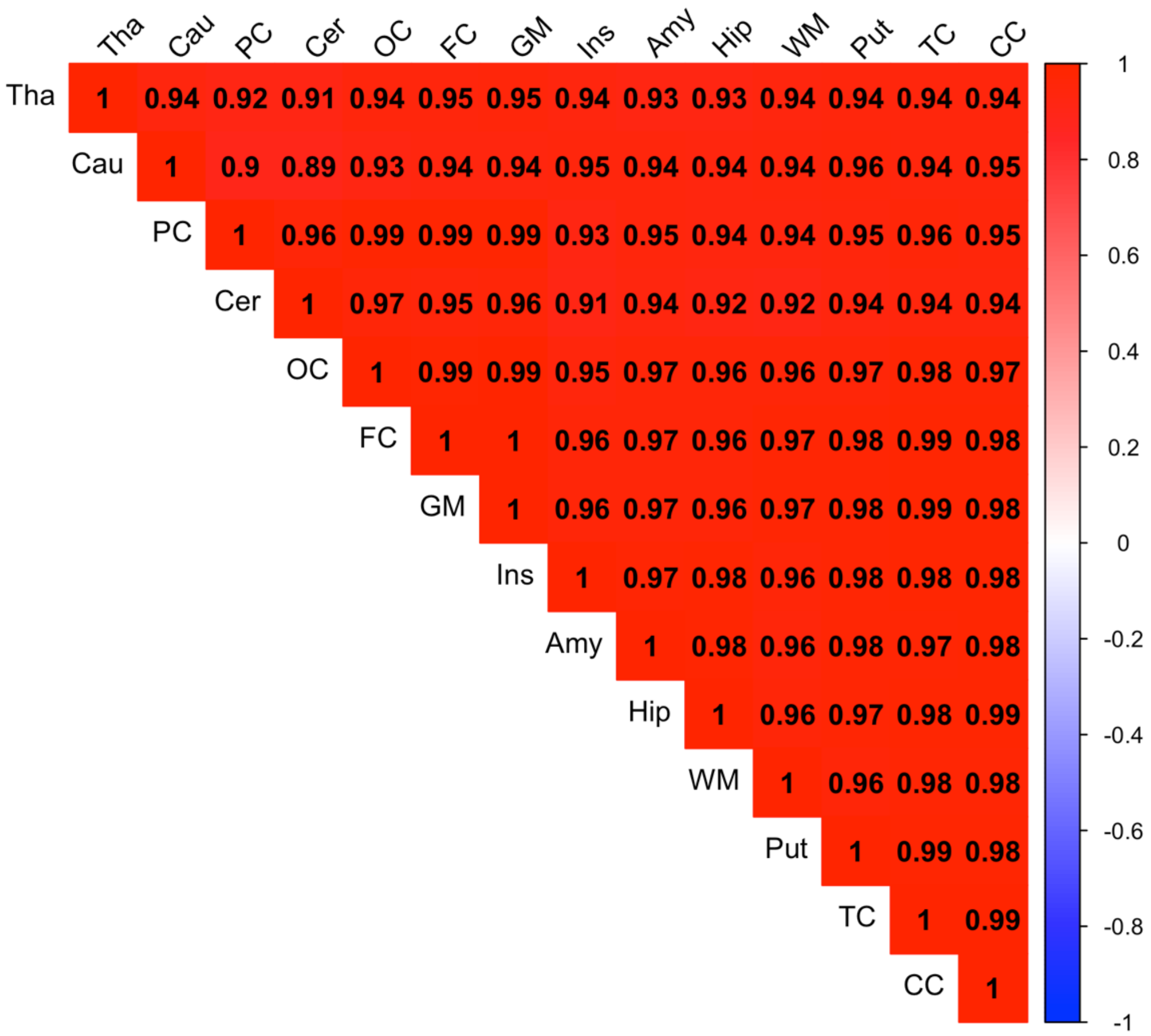

Figure S1. Pearson correlations between regional CB1 receptor availability values. Tha = Thalamus, Cau = caudate, PC = parietal cortex, Cer = cerebellum, OC = occipital cortex, FC = frontal cortex, GM = grey matter, Ins = insulin, Amy = amygdala, Hip = hippocampus, WM = white matter, Put = putatmen, TC = temporal cortex, CC = cingulate cortex.

Table S1. Mean and standard deviation of CB1 receptor availability values.

ROIs	Mean	Standard deviation
Frontal cortex	14.13	3.98
Temporal cortex	14.35	3.90
Pariatal cortex	14.33	4.29
Occipital cortex	12.41	3.40
Cingulate cortex	14.19	3.99
Putamen	15.99	4.42
Thalamus	8.02	1.85
Amygdala	12.02	3.45
Insulin	14.66	4.00
Caudate	10.82	2.95
Hippocampus	10.37	2.69
Cerebellum	10.20	2.61
Grey matter	13.90	3.88
White matter	7.44	1.99
